# A Fluorescent Aptasensor Based on Assembled G-Quadruplex and Thioflavin T for the Detection of Biomarker VEGF165

**DOI:** 10.3389/fbioe.2021.764123

**Published:** 2021-11-16

**Authors:** Xin Zheng, Shunxiang Gao, Jihong Wu, Xiaobo Hu

**Affiliations:** ^1^ Department of Clinical Laboratory, Longhua Hospital, Shanghai University of Traditional Chinese Medicine, Shanghai, China; ^2^ Eye Institute and Department of Ophthalmology, Eye and ENT Hospital, Fudan University, Shanghai, China; ^3^ Institutes of Brain Science, State Key Laboratory of Medical Neurobiology and MOE Frontiers Center for Brain Science, Fudan University, Shanghai, China; ^4^ NHC Key Laboratory of Myopia, Fudan University, Shanghai, China; ^5^ Key Laboratory of Myopia, Chinese Academy of Medical Sciences, Shanghai, China; ^6^ Shanghai Key Laboratory of Visual Impairment and Restoration, Shanghai, China

**Keywords:** aptamer, biomarker, biosensor, assembled G-quadruplex, thioflavin T

## Abstract

VEGF165, a regulator of angiogenesis, has been widely used as a serum biomarker for a number of human diseases, including cancer, rheumatoid arthritis, bronchial asthma, and diabetic eye disease. The rapid, accurate, and convenient detection of VEGF165 is a crucial step in effective healthcare monitoring, disease diagnosis, and prognosis assessment. In this study, a fluorescent aptasensor based on an assembled G-quadruplex and the signal molecule ThT was developed for VEGF165 detection. First, G-rich DNA fragments were assembled at both ends of the anti-VEGF165 aptamer, and the B-DNA form was converted into a G-quadruplex structure aptamer (G4-Apt). Then, ThT was introduced, and the G-quadruplex significantly enhanced the fluorescence intensity of the bound ThT. When VEGF165 was present, the higher affinity of the aptamer to the target protein allowed the G4-Apt/VEGF165 complex to form and release ThT, which emitted only weak fluorescence in the free state. Therefore, the aptasensor exhibited a good linear detection window from 1.56 to 25 nM VEGF165, with a limit of detection of 0.138 nM. In addition, the aptasensor was applied to detect VEGF165 in clinical serum samples, showing good accuracy, reproducibility, and stability. These results indicate that our developed fluorescent aptasensor can potentially be a reliable, convenient, and cost-effective approach for the sensitive, specific, and rapid detection of the VEGF165 biomarker.

## Introduction

Vascular endothelial growth factor (VEGF) is a strong endothelial-cell-specific mitogen that plays central roles in the regulation of physiologic and pathologic angiogenesis (Greenberg and Jin, 2004). It can bind to extracellular receptor tyrosine kinases and activate different intracellular signaling pathways, which will in turn stimulate vascular endothelial cell growth, survival, and proliferation (Olsson et al., 2006). In mammals, the VEGF family consists of five subgroups: VEGF-A, VEGF-B, VEGF-C, VEGF-D, and the placenta growth factor (Goel and Mercurio, 2013; Simons et al., 2016; Rud’ko et al., 2017). VEGF-A is the prototype member, with at least four isoforms: VEGF121, VEGF165, VEGF189, and VEGF206. Among these, VEGF165 is the most potent pro-angiogenic isoform and has been widely used as a biomarker for several human diseases, including cancer, rheumatoid arthritis, bronchial asthma, and diabetic eye disease (Nakahara et al., 2003; Niu and Chen, 2010; Paradowska-Gorycka et al., 2015; Tsai et al., 2015; Wong et al., 2016). Moreover, VEGF165 levels also significantly differ in various stages of human disease. For example, the serum of patients with diabetes, non-proliferative diabetic retinopathy, and proliferative diabetic retinopathy contains higher levels of VEGF165 compared with serum from healthy individuals (Manaviat et al., 2011; Ahuja et al., 2019; Abu-Yaghi et al., 2020; Wang et al., 2020). Therefore, accurate, sensitive, and rapid detection of the disease-specific biomarker VEGF165 in bodily fluids may serve as an effective tool for healthcare monitoring as well as disease diagnosis and prognosis.

Currently, various VEGF165 detection techniques based on immunoassays have been reported, including immunohistochemistry, radioimmunoassays, and enzyme-linked immunosorbent assays (ELISAs) (Anthony et al., 1997; Goncalves et al., 2007; Takahashi et al., 2016). Although these traditional immunological methods provide invaluable insights for the analysis of VEGF165, they are labor-intensive and time-consuming and require cumbersome operating protocols, which limit their application in real-time clinical diagnosis. In recent years, aptamer-based biosensors have gained strong clinical application prospects for the detection of disease-specific biomarkers (Zhang et al., 2018; Gao S. et al., 2020; Chinnappan et al., 2021; Gao et al., 2021; Liu et al., 2021; Xu et al., 2021). An aptamer, defined as a chemical antibody, is a functional single-stranded DNA or RNA oligonucleotide that can bind to a target with high affinity and specificity (Ellington and Szostak, 1990; Tuerk and Gold, 1990). It can be obtained by *in vitro* SELEX technology and folded into a unique three-dimensional structure, such as G-quadruplex, B-DNA, Z-DNA, and i-motif, to perform its recognition, diagnosis, and treatment functions (Yuan et al., 2017; Gao L. et al., 2020; Hassani et al., 2020; Manibalan et al., 2020; Xu et al., 2020; Li et al., 2021). Compared with antibodies, aptamers have significant advantages as novel recognition molecules (Radom et al., 2013; Vigneshvar et al., 2016; Zhou and Rossi, 2017). For example, they have a wide range of targets, low immunogenicity, and low toxicity. They can also be prepared by chemical synthesis in large batches without the need to immunize animals. Additionally, aptamers are easy to modify and label, inexpensive, and less affected by environmental factors, such as pH and temperature. This allows for easy long-term storage and transportation. Because of these advantages, many aptasensors based on surface plasmon resonance, surface-enhanced Raman scattering, and electrochemical workstations have been reported for VEGF165 detection (Zhao et al., 2015; Xu et al., 2017; Dehghani et al., 2018; Fu et al., 2020; Lee et al., 2020). However, expensive sensing equipment, complicated operation procedures, and high reliance on professional operators are the main hindrances that limit their point-of-care testing in clinical applications.

In this study, a simple, fast, accurate, and cost-effective aptasensor was established for VEGF165 detection using an assembled G-quadruplex and the signal molecule thioflavin T (ThT). First, the anti-VEGF165 aptamer with a B-DNA form was successfully assembled into a G-quadruplex structure aptamer (G4-Apt). Then, the fluorescent dye ThT was introduced, which acts as a signal indicator by specifically binding to the G-quadruplex. The G-quadruplex could significantly enhance the fluorescence intensity of the bound ThT. When VEGF165 was present, the higher affinity of the aptamer to the target protein promoted the transformation of the G4-Apt/ThT to the G4-Apt/VEGF165 complex. The bound ThT was simultaneously released, and it emitted only weak fluorescence in the free state. Therefore, the developed fluorescent aptasensor exhibited a linear detection window, from 1.56 to 25 nM; good sensitivity, with a limit of detection (LOD) of 0.138 nM; and a high target specificity for VEGF165. Furthermore, the aptasensor was applied to detect VEGF165 in spiked serum samples. A recovery percentage of 95.33–109.18% and a coefficient of variation of 3.39–7.69% were obtained, suggesting that the proposed detection system has good accuracy, reproducibility, and stability. Therefore, the developed aptasensor offers a reliable, convenient, and cost-effective approach for the sensitive, specific, and rapid detection of the VEGF165 biomarker.

## Materials and Methods

### Materials and Reagents

The anti-VEGF165 sequence (aptamer: 5′-CCG​TCT​TCC​AGA​CAA​GAG​TGC​AGG​G -3′) and the assembled sequence (G4-Apt: 5′-AGG​GAC​GGG​ACC​GTC​TTC​CAG​ACA​AGA​GTG​CAG​GGA​GGG​ACG​GGA-3′) in this study were synthesized by Sangon Biotech Co., Ltd. (Shanghai, China). Recombinant human VEGF165 protein and VEGF165 ELISA kits were purchased from Sino Biological Inc. (Beijing, China). Human serum albumin (HSA), streptavidin (SA), bovine serum albumin (BSA), adenosine triphosphate (ATP), and thioflavin T (ThT) were obtained from Sigma-Aldrich Co. LLC (United States). Gliotoxin (GTX) was purchased from Puhuashi Technology Development Co., Ltd. (Beijing, China). SA biosensors were obtained from ForteBio (Menlo Park, CA). All solutions were prepared using Milli-Q ultrapure water (Millipore, Billerica, MA).

### Binding Affinity Determination by Biolayer Interferometry

In this study, the binding affinity of the aptamer sequence was measured using biolayer interferometry in an Octet RED96 system (ForteBio, Shanghai). The principle and analysis procedures were detailed in our previously reported protocol (Gao et al., 2019b; Gao et al., 2019c). All steps were performed at 25°C with shaking at 1,000 rpm in a 96-well plate containing 200 μL of binding buffer in each well. The response data obtained from the reaction surface were normalized by subtracting the signal simultaneously acquired from the reference surface to eliminate nonspecific binding and a buffer-induced interferometry spectrum shift using Octet Data Analysis Software CFR Part 11 Version 10.0.0.5. Finally, the affinity parameter, *K*_*D*_, was obtained.

### Conformation Analysis by Circular Dichroism

The conformations of the aptamer and G4-Apt were analyzed using a Chirascan™ V100 Circular Dichroism (CD) Spectrometer. Before the assay, the chamber was deoxygenated with dry purified nitrogen (99.99%) and kept in the nitrogen atmosphere during experiments. Each CD spectrum was collected from 320 to 220 nm, representing the accumulation of two scans at 50 nm/min, with a 1-nm bandwidth and a time-per-point of 0.5 s.

### VEGF165 Detection by the Fluorescent Aptasensor

This study was approved by the Ethics Committee of the Eye and ENT Hospital of Fudan University, and consent was obtained from all participants. Aptasensor preparation and VEGF165 detection were conducted according to the following procedure. G4-Apt was diluted to a concentration of 2.5 μM and then heated to 95 °C for 10 min, cooled in an ice bath for 5 min, and kept at room temperature for 10 min. The 25 μL of each aliquot G4-Apt was added to 50 μL of ThT (5 μM), and the mixture was incubated for 30 min. Subsequently, 25 μL of VEGF165 at different concentrations was added, and the mixture was incubated at room temperature for another 40 min. Finally, the fluorescence spectra were recorded immediately by a Spark® Multimode Microplate Reader (Tecan Trading AG) at 25°C. The fluorescence emission spectra of the developed detection system were monitored from 450 to 600 nm, with an excitation wavelength of 390 nm. Fluorescence intensity at 490 nm was used for quantitative analysis.

## Results and DISCUSSION

### Construction and Characterization of G-Quadruplex Structure

**FIGURE 1:**
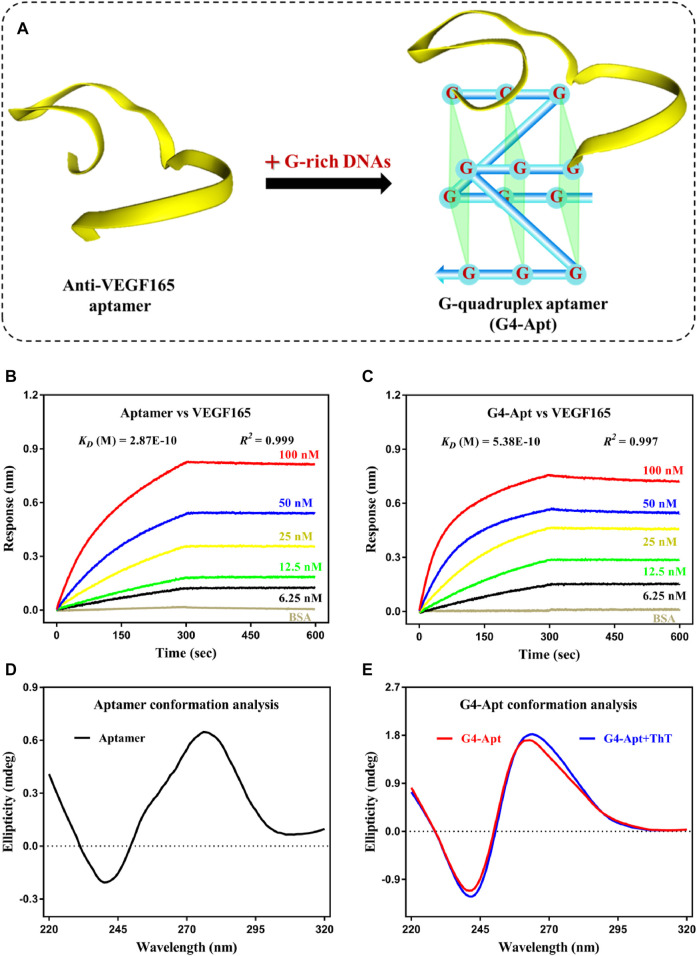
**(A)** Schematic diagram of the construction of the G-quadruplex aptamer. **(B,C)** Affinity and specificity analysis of the anti-VEGF165 aptamer and the assembled G4-Apt by biolayer interferometry. **(D,E)** Conformation analysis of the anti-VEGF165 aptamer and the assembled G4-Apt by circular dichroism.

As shown in Figure 1A, G-rich DNA fragments (AGGGACGGGA) were assembled at both ends of the anti-VEGF165 aptamer to construct a G-quadruplex structure aptamer (G4-Apt) (Potty et al., 2009; Gao H. et al., 2019; Gao et al., 2019c). The binding ability of the aptamer and assembled G4-Apt was first evaluated by biolayer interferometry (BLI). As shown in Figures 1B,C, various concentrations of VEGF165 (100, 50, 25, 12.5, and 6.25 nM) were analyzed for both an association time and a dissociation time of over 5 min, along with a blank sample containing only BSA for reference. The BLI results suggest that the aptamer and G4-Apt can strongly bind to VEGF165, with binding affinities of 0.287 and 0.538 nM, respectively. Although the aptamer was modified using G-rich DNA fragments, the G4-Apt maintained a consistent strong affinity and specificity for VEGF165. To further analyze the conformational changes, the tertiary structures of the aptamer and G4-Apt were determined by circular dichroism (CD). In Figure 1D, the CD spectrum shows that the aptamer folded into a B-DNA structure according to a positive peak at 276 nm and a negative peak at 240 nm. Compared with the CD spectrum of the aptamer, the G4-Apt largely possessed a parallel G-quadruplex structure, with a characteristic positive peak at 262 nm and a negative peak at 240 nm (Figure 1E). Therefore, an anti-VEGF165 aptamer with a B-DNA form was successfully assembled into a G-quadruplex structure that maintained binding ability at the sub-nanomolar level.

### Detection Mechanism of the Fluorescent Aptasensor

**FIGURE 2:**
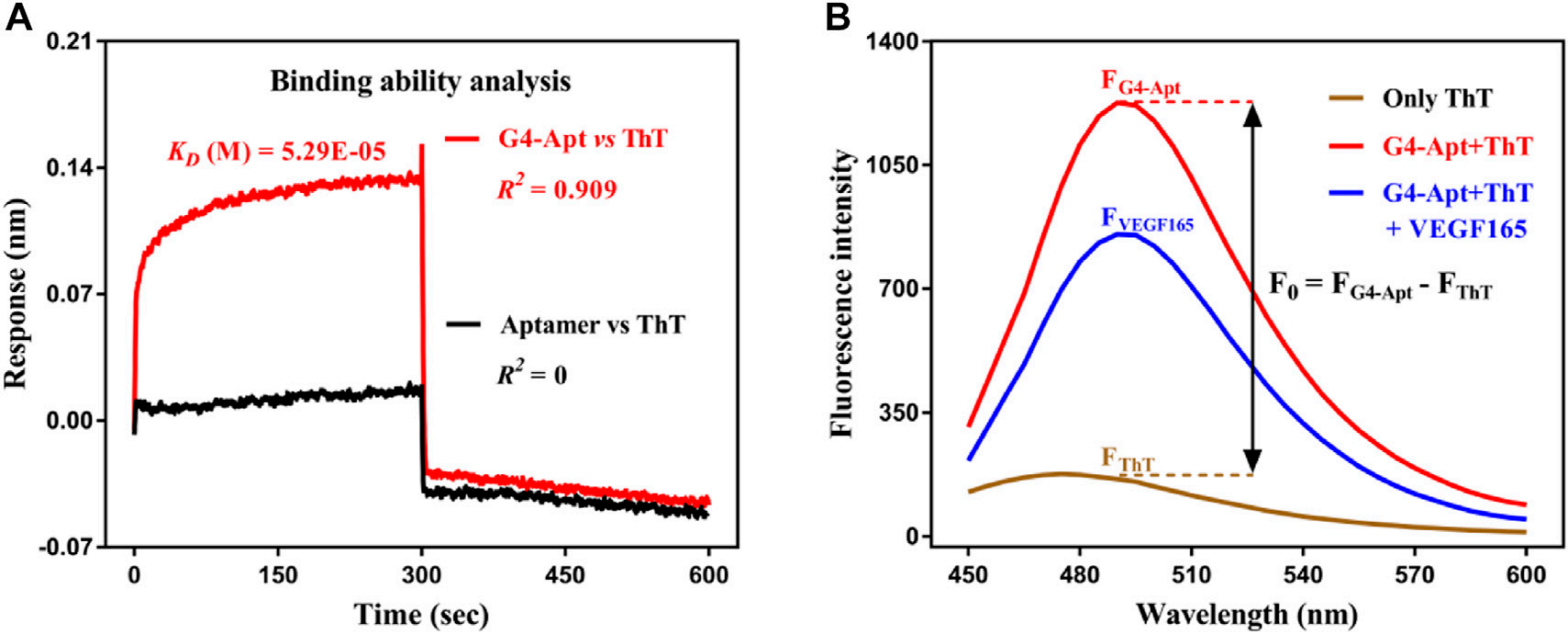
**(A)** Binding affinity analysis of the assembled G4-Apt and the aptamer to thioflavin T (ThT) by biolayer interferometry. **(B)** Fluorescence spectra of ThT in the free state (brown curve), ThT with G4-Apt (red curve), and ThT with G4-Apt and VEGF165 (blue curve).

ThT is a commercially available benzothiazole cationic dye with good chemical stability, ideal water solubility, excellent discrimination, and a low-fluorescence background in the free state. It exhibits highly specific interactions with G-quadruplexes through intercalation, groove-binding, and end stacking, and further leads to a significant fluorescence enhancement effect (Mohanty et al., 2013; de la Faverie et al., 2014). On this basis, ThT was introduced in this study as a signal indicator for construction of the aptasensor. The BLI interaction results show that ThT did not bind to the aptamer (Figure 2A) and had a moderately applicable binding force to G4-Apt (*K*_*D*_ = 52.9 μM). Obviously, this is significantly lower than the binding affinity of G4-Apt to VEGF165 (*K*_*D*_ = 0.538 nM). The CD spectrum suggests that the intensity and width of the characteristic absorption peak of G4-Apt increased in the presence of ThT (Figure 1E). These results indicate that ThT can not only specifically bind to G4-Apt but may also induce the formation of more G-quadruplex structures. Therefore, ThT was used as an ideal fluorescent signal indicator to construct the following detection system.

**FIGURE 3:**
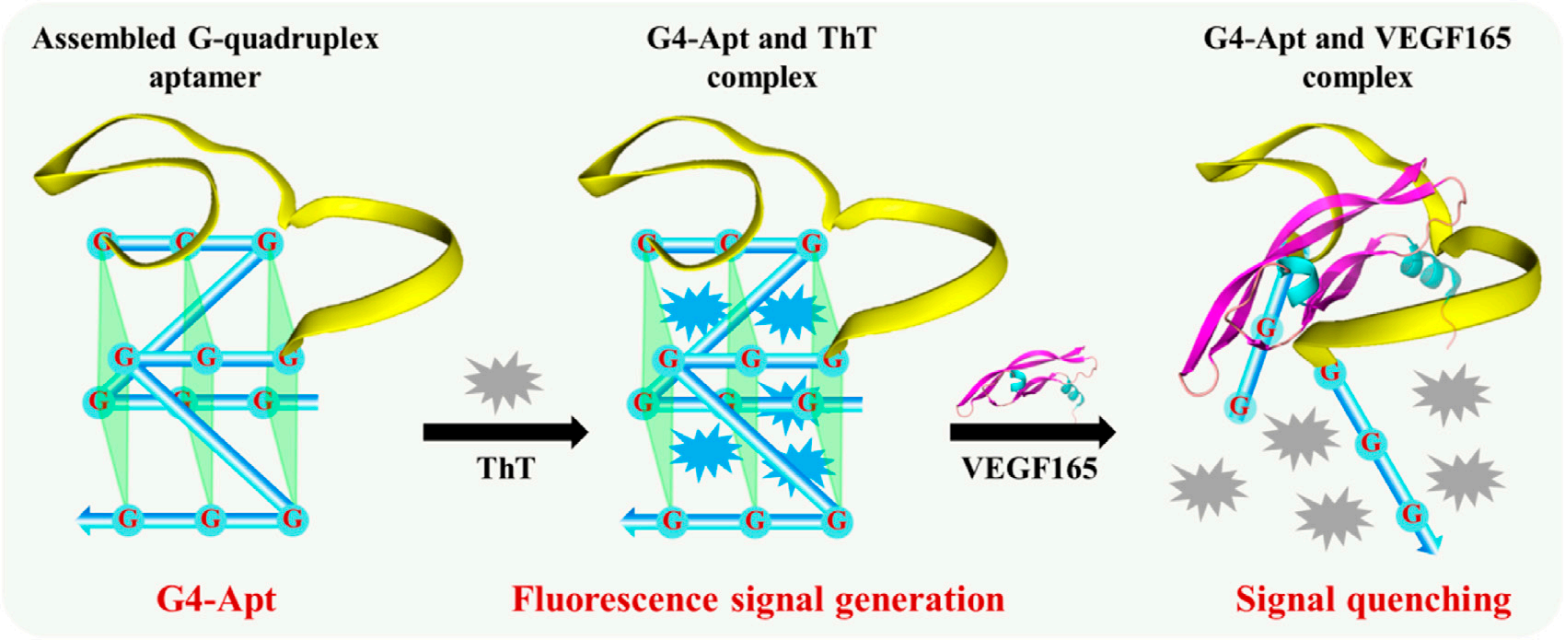
Illustration of the developed fluorescent aptasensor based on the assembled G-quadruplex aptamer and thioflavin T (ThT).

To achieve rapid, sensitive, and effective detection of the VEGF165 biomarker, a fluorescent aptasensor based on the assembled G-quadruplex and ThT was developed. As shown in Figure 3, the G-rich DNA fragments assembled at both ends of the anti-VEGF165 aptamer formed the parallel G-quadruplex aptamer G4-Apt. When ThT was present, its fluorescence intensity was substantially enhanced through specific intercalation into the G-quadruplex (Figure 2B), while ThT emitted only weak fluorescence in the free state. When VEGF165 was added, because of the higher affinity of the aptamer to the target protein, it promoted the structural transformation of G4-Apt and resulted in loss of the G-quadruplex structure. The G4-Apt/VEGF165 binding complex was further formed, and ThT was released (Figure 3), which led to a significant decrease in the fluorescence intensity of the detection system (Figure 2B). Therefore, the efficiency of the change in the fluorescence intensity during this process could be used for detecting VEGF165.

### Parameter Optimization of the Fluorescent Aptasensor

**FIGURE 4:**
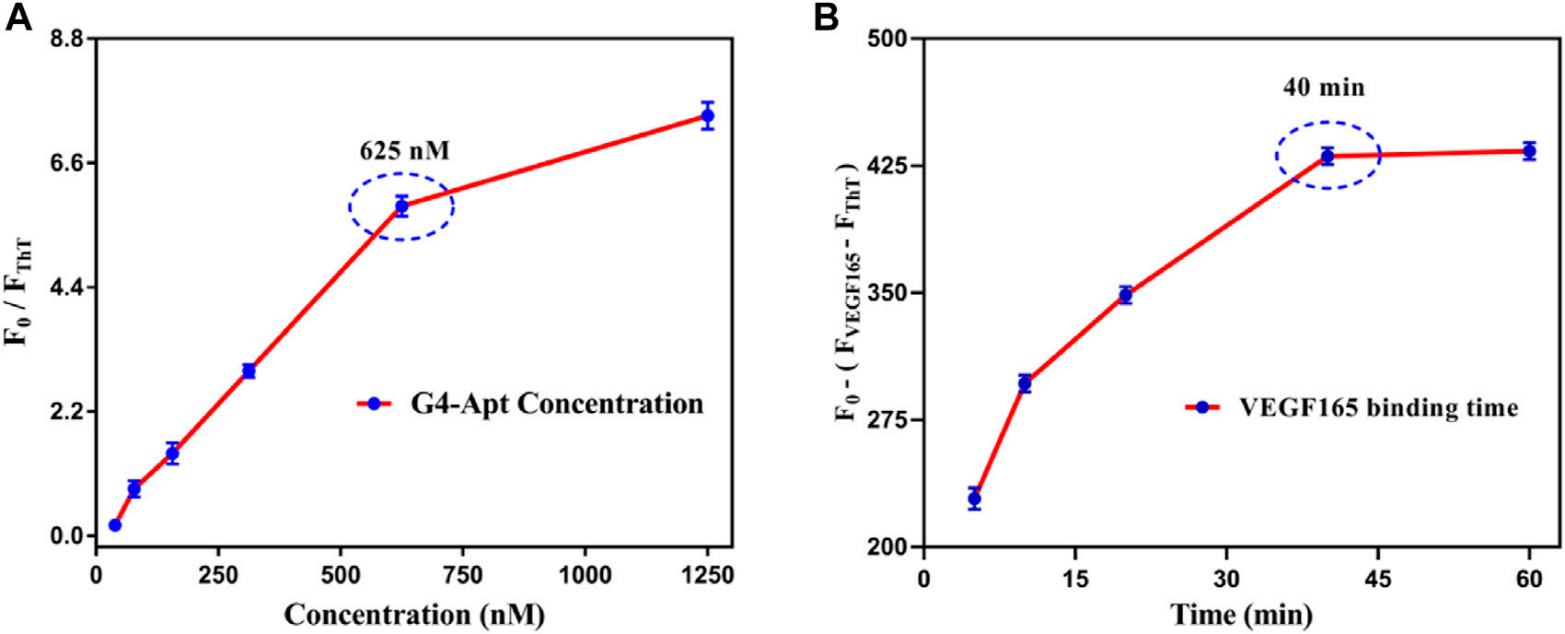
**(A)** Optimization of the concentration for the assembled G4-Apt. **(B)** Optimization of binding time for VEGF165 in the detection system.

To achieve the best detection efficiency of the fluorescent aptasensor, the concentration ratio of the G4-Apt to ThT was first studied in the detection system. Theoretically, a higher concentration of G4-Apt (high ratio) will result in a decrease in detection sensitivity, while a lower G4-Apt concentration will not have enough detection windows. To balance this effect, the optimal G4-Apt to ThT ratio was analyzed by titrating the G4-Apt concentration (ranging from 39.06 to 1,250 nM) with a fixed concentration of ThT. As shown in Figure 4A, as the G4-Apt concentration continued to increase, the available detection fluorescence also increased significantly. A turning point occurred when the G4-Apt concentration was 625 nM. Although a higher concentration of G4-Apt could produce a wider detection window, it might also greatly reduce the detection sensitivity. Therefore, a concentration of 625 nM G4-Apt was selected to balance the detection window and the sensitivity of the fluorescent aptasensor. In addition, it is important that the detection time allows VEGF165 to induce the structural conversion of the G-quadruplex and specifically bind to the aptamer to form the stable detection system. Generally, a longer detection time is conducive to the formation of a highly stable complex for a greater fluorescence change effect. However, a longer detection time will also reduce the application prospects of the aptasensor. To balance this influence, a binding time ranging from 5 to 60 min was evaluated. As shown in Figure 4B, the intensity of fluorescence change increased with higher VEGF165 incubation times, reaching equilibrium at 40 min. Therefore, 40 min was selected as the optimal detection time for VEGF165 in the following experiments.

### Detection Range, Sensitivity, and Specificity of the Fluorescent Aptasensor

**FIGURE 5:**
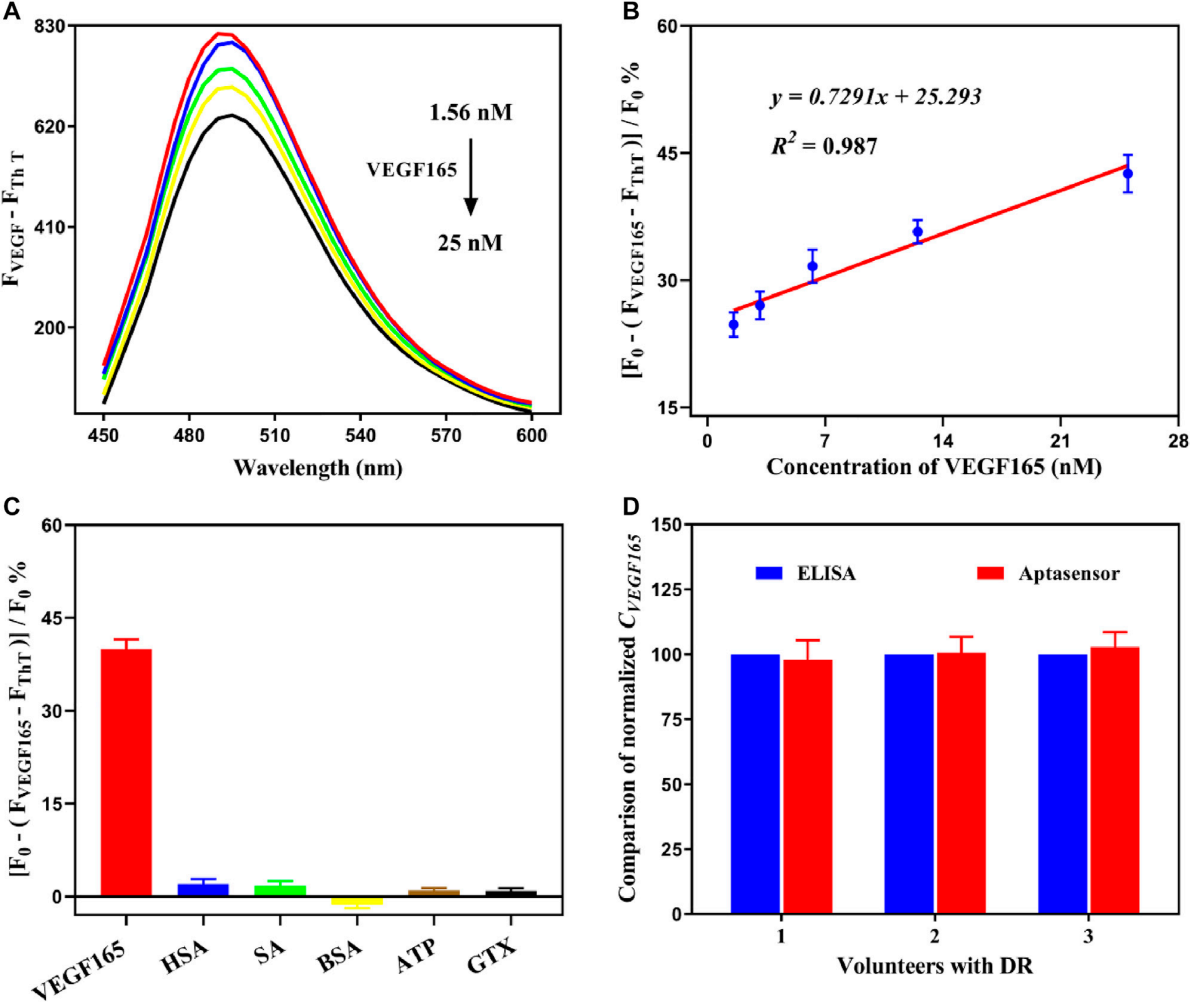
**(A)** Fluorescence spectra of the detection system at various concentrations of VEGF165. **(B)** Linear relationship between the aptasensor signal and the concentration of VEGF165 in the range of 1.56–25 nM. **(C)** Cross-reactivity experiments of the fluorescent aptasensor with HAS, SA, BSA, ATP, and GTX. The error bars represent the standard deviation of three independent measurements. **(D)** Comparison of the normalized concentration of VEGF165.

To further evaluate the detection of VEGF165 using the fluorescent aptasensor, dose-dependent changes in the signal range of 1.56–25 nM were investigated under optimal experimental conditions. As shown in Figure 5A, the intensity of the fluorescence spectrum gradually decreased in the presence of VEGF165. The aptasensor signal was quantified as y= (F_0_–(F_*VEGF165*_–F_*ThT*_)]/F_0_% and plotted against the VEGF165 concentration (Figure 5B), with each sample analyzed in triplicate. Subsequently, a good linear relationship was obtained in the concentration range from 1.56 to 25 nM, which can be represented by the linear regression: y=0.7291x+25.293 , *R* ^*2*^ = 0.987, with an LOD of 0.138 nM. The LOD was calculated based on the *3*σ*/k* rule, where *σ* is the standard deviation of the aptasensor signal in the absence of the analyte (*n* = 12), and *k* is the slope of the linear equation. As specificity is an important performance parameter of an aptasensor in practical applications, cross-reactivity experiments were conducted using several markers as interfering substances, including HAS, SA, BSA, ATP, and GTX. As shown in Figure 5C, after adding equal concentrations of other markers, low aptasensor responses were observed. This contrasted with the high signal triggered by VEGF165. Therefore, the target protein could be detected with high specificity by the aptasensor. These results demonstrate that our developed fluorescent aptasensor can realize the sensitive, specific, and rapid detection of the VEGF165 biomarker.

### Detection of VEGF165 in Serum Samples by the Fluorescent Aptasensor

To further verify the effectiveness of our developed fluorescent aptasensor, free VEGF165 was added to the clinical serum sample and analyzed using the preset calibration curve. A good recovery percentage of 95.33–109.18% was obtained, indicating that the detection accuracy of the aptasensor was not significantly affected by clinical serum samples. The coefficient of variation range of 3.39–7.69% was acceptable (less than 10%), suggesting that the proposed detection system has high reproducibility and stability. In addition, the concentrated serum samples, which were obtained from diabetic retinopathy (DR) patients, were simultaneously analyzed using an ELISA kit and the fluorescent aptasensor. As shown in Figure 5D, the results were highly consistent, which indicates that our developed aptasensor can be reliably applied to actual sample analysis. Therefore, the developed fluorescent aptasensor has significant potential as a reliable, convenient, and cost-effective approach for the sensitive, specific, and rapid detection of the VEGF165 biomarker.

## Conclusion

In this study, a reliable, convenient, and cost-effective aptasensor was established for the sensitive, specific, and rapid detection of VEGF165 biomarker using an assembled G-quadruplex and signal molecule ThT. The developed fluorescent aptasensor shows reliable diagnostic performance. Its sub-nanomolar LOD can provide sufficiently high sensitivity levels comparable to ELISA. Although ELISA is the most common method for biomarker detection, it requires at least 3 h to complete VEGF165 analysis, whereas our fluorescent aptasensor can be used to complete this quantification in about 1 h. Further comparisons between this work and previously reported aptasensors for analyzing VEGF165 are shown in Table 1. Compared with fluorescence-based aptasensors (Wang and Si, 2013; Wang et al., 2014), although they have similar detection windows and sensitivity, but the construction scheme of our aptasensor has universal structures that can broadly extend the development and application of biosensors based on the assembled G-quadruplex. Compared with sensors based on colorimetric, electrochemical, and surface plasmon resonance methodologies (Chen et al., 2014; Pan et al., 2017; Dong et al., 2020), the aptasensor is still insufficient in automation, visualization, and sensitivity. However, our developed aptasensor does not require enzyme labeling or rely on expensive equipment and professional operators. Its simple, convenient, and fast detection process makes it particularly suitable for the point-of-care testing needed for disease biomarkers. We believe that this aptasensor design may soon promote its clinical translational research in the future.

**TABLE 1:** Comparison of different aptamer-based detection methods for VEGF165.

Detection strategy	Range	LOD	Reference
Aptasensor based on fluorescence polarization	0.32–5 nM	0.32 nM	Wang et al. (2014)
Aptasensor based on graphene oxide fluorescence resonance energy transfer	0.5–5 nM	0.25 nM	Wang and Si (2013)
Colorimetric aptasensor	100–1 × 10^5^ pg/ml	10 pg/ml	Dong et al. (2020)
Electrochemical aptasensor	50–1 × 10^5^ pg/ml	50 pg/ml	Pan et al. (2017)
Surface plasmon resonance Aptasensor	10–1 × 10^6^ pg/ml	100 pg/ml	Chen et al. (2014)
Aptasensor based on assembled G-quadruplex and Thioflavin T	1.56–25 nM	0.138 nM	This work

## Data Availability

The raw data supporting the conclusion of this article will be made available by the authors, without undue reservation.
